# Periodontal Pathogens and Adverse Pregnancy Outcomes: A Narrative Review

**DOI:** 10.3390/life13071559

**Published:** 2023-07-13

**Authors:** Mishali AlSharief, Esraa Alabdurubalnabi

**Affiliations:** 1 Department of Preventive Dental Sciences, College of Dentistry, Imam Abdulrahman Bin Faisal University, P.O. Box 1982, Dammam 31441, Saudi Arabia; 2 Fellowship in Periodontics Program, College of Dentistry, Imam Abdulrahman Bin Faisal University, P.O. Box 1982, Dammam 31441, Saudi Arabia

**Keywords:** periodontitis, periodontal pathogens, oral microbiome, inflammatory mediators, pregnancy, adverse pregnancy outcomes, preterm births, low birth weight

## Abstract

Periodontal disease is a multi-microbial infection of the teeth-supporting apparatus that manifests as clinical attachment loss and alveolar bone loss. The association between periodontal disease and systemic diseases has been proposed in the literature owing to the former’s chronic state of inflammation, and adverse pregnancy outcomes are no exception. As a result of periodontal pathogen invasion, a series of systemic inflammatory and immunologic events affecting the safety of the fetoplacental unit may unfold. This may be further exaggerated by physiologic hormonal and metabolic fluctuations during pregnancy. This can not only negatively affect the gestation period and consequently cause preterm low weight but also complicate the pregnancy via preeclampsia and gestational diabetes. This narrative review article aims to provide a summary of relevant available evidence pertinent to the relationship between periodontal diseases, associated periodontal pathogens and virulence mechanisms mediated by pro-inflammatory cytokines and prostaglandins, and adverse pregnancy outcomes. Furthermore, this article highlights some of the literature addressing the impact of periodontal therapy interventions and pregnancy outcomes.

## 1. Introduction

The oral cavity is a complex environment, providing an optimal habitat for the growth of the most diverse host-compatible microorganisms in the human body. This interactive relationship between the host and microbiota is called symbiosis. Any disruption in this relationship as a result of environmental or host-response-related factors will result in unfavorable dysbiosis and the subsequent initiation and progression of destructive periodontal diseases [1]. While the direct catastrophic outcomes of dysbiosis have been shown in the local oral environment, its impact is yet to be systemically studied and is a trending topic of investigation.

Periodontitis is a chronic inflammatory disease that destroys the dental attachment apparatus, including gingival epithelial, connective tissues, and the surrounding alveolar housing. Signs and symptoms include increased periodontal pocket depth, bleeding upon probing, clinical attachment loss, and alveolar bone loss as a result of progressive periodontal destruction. If left untreated, periodontitis can worsen, leading to tooth mobility, occlusal trauma, pathologic migration, and eventually tooth loss [2]. Setting aside the genetic component of this disease, factors such as the continuously changing nature of the oral environment—including shifts in pH levels, skewed temperature, and continuous nutrient intake—and metabolism throughout the day can drastically disturb symbiosis in oral microorganisms, creating an optimal environment for pathogenic, Gram-negative anaerobic bacterial colonization and multiplication [2]. In combination with other modifying factors, such as nutritional deficiencies, medications, increased stress levels, hormonal changes during pregnancy or adolescence, and smoking habits, dysbiotic dental biofilm was identified as a primary etiologic risk factor for the initiation and progression of periodontal diseases [3]. As the disease progresses, based on the host response status, the immune system may eventually fail to eradicate the causative bacterial load, and this is when tissue destruction and infection dissemination occur locally and systemically [4].

Recently, studies found an influential association between the pathophysiology of periodontitis and systemic diseases such as diabetes mellitus, cardiovascular diseases, and adverse pregnancy outcomes [5]. A two-way relationship was established between diabetes and periodontitis in that controlling or exacerbating either disease’s clinical parameters can have a positive or negative impact on the progression of the other. The pathogenic bacteria responsible for periodontitis, such as *Porphyromonas gingivalis* and *Prevotella intermedia*, were found to be significantly higher in patients suffering from type 1 diabetes mellitus when compared with periodontally healthy controls [5]. Furthermore, it was concluded that increased severity in periodontal diseases is followed by poor glycemic control (HbA1C levels > 9%) in diabetic patients [6]. On the other hand, neutrophil function impairment, defective collagen production and degradation, and altered receptor activator in the NF-kB ligand and osteoprotegerin (RANKL:OPG) ratio in diabetic patients serve as mechanisms aggravating periodontal pathogen destruction in tooth-supporting tissues [7]. The multi-pathogenicity of periodontal disease alters systemic responses, potentially influencing the host’s cardiovascular health. Hasturk et al. recently demonstrated the systemic influence of periodontal infection, specifically *P. gingivalis*, on an atherosclerotic animal model and found that treating periodontal diseases is beneficial in reducing systemic inflammatory infiltrate, lowering circulating C-reactive protein, and eventually arresting/interrupting vascular inflammation and atheroma formation [8].

Even today, preterm low birth weight (PLBW) is a significant public health issue. Infants affected are at higher risk of respiratory distress syndrome, cerebral palsy, pathologic heart conditions, epilepsy, and severe learning problems [9]. In 1976, PLBW was defined as a birth weight of less than 2500 g with a gestational age of less than 37 weeks by the 29th World Health Assembly. The cause of low birth weight is often unclear. A quarter to half of all PLBW deliveries occur because of an unknown etiology [10]. In most cases, this may result directly from a short gestational period and/or retarded intrauterine growth. Although smoking and low socioeconomic status are linked to PLBW [11], other risk factors may include genetic features, alcohol consumption, maternal malnutrition, poor prenatal care, and urinary tract infections [12]. Several epidemiologic studies have investigated and targeted PLBW with public health interventions [13]. The rate of preterm birth appears to be increasing in the Western world despite a better understanding of reproductive physiology and pharmacological advances that can arrest preterm labor [14]. Furthermore, smoking has been identified as a major risk factor for both preterm birth and periodontal diseases; however, a study conducted by Skuldbol et al. revealed contradictory results, finding a lack of association between preterm birth and both periodontitis and smoking [15]. A possible justification for these results could be that it is uncommon for women of childbearing age to suffer periodontitis.

Other adverse pregnancy outcomes include pregnancy-induced hypertension or preeclampsia (a critical condition characterized by maternal hypertensive episodes, along with proteinuria and edema), miscarriage (the birth of a baby before completing 6 months of gestation with no signs of life), stillbirths (the birth of a baby after completing 6 months of gestation with no signs of life), premature ruptures of fetal membranes, gestational diabetes mellitus, and others [16]. This literature review focused on PLBW, as it is one of the most reported pregnancy complications and is well explored in the literature.

This narrative review article aims to provide a summary of relevant available evidence pertinent to the relationship between periodontal diseases, associated periodontal pathogens and virulence mechanisms, and adverse pregnancy outcomes. Furthermore, this article highlights some of the literature addressing the impact of periodontal therapy interventions and pregnancy outcomes.

## 2. Materials and Methods

### 2.1. PICO Questions

To address the aim of this review, focused PICO questions were constructed as follows:

1. Are pregnant women with periodontitis at greater risk of developing adverse pregnancy outcomes in comparison with periodontally healthy women?
2. Within the available literature, what is the relationship between periodontal diseases and adverse pregnancy outcomes?
3. In pregnant women with periodontitis, what is the role of periodontal pathogens and pro-inflammatory mediators in increasing the risk of preterm low birth weight?
4. In pregnant women with periodontitis, does periodontal therapy during the gestational period reduce the risk of adverse pregnancy outcomes compared with no intervention?

### 2.2. Selection Criteria

This review includes articles with the following criteria:

- Originally published in English;
- Available in full text;
- Articles addressing preterm births (PTBs) and low birth weight (LBW) as adverse pregnancy outcomes.

Articles published in languages other than English or discussing pregnancy outcomes other than PTBs or LBW were excluded.

### 2.3. Search Methodology

**Figure 1:**
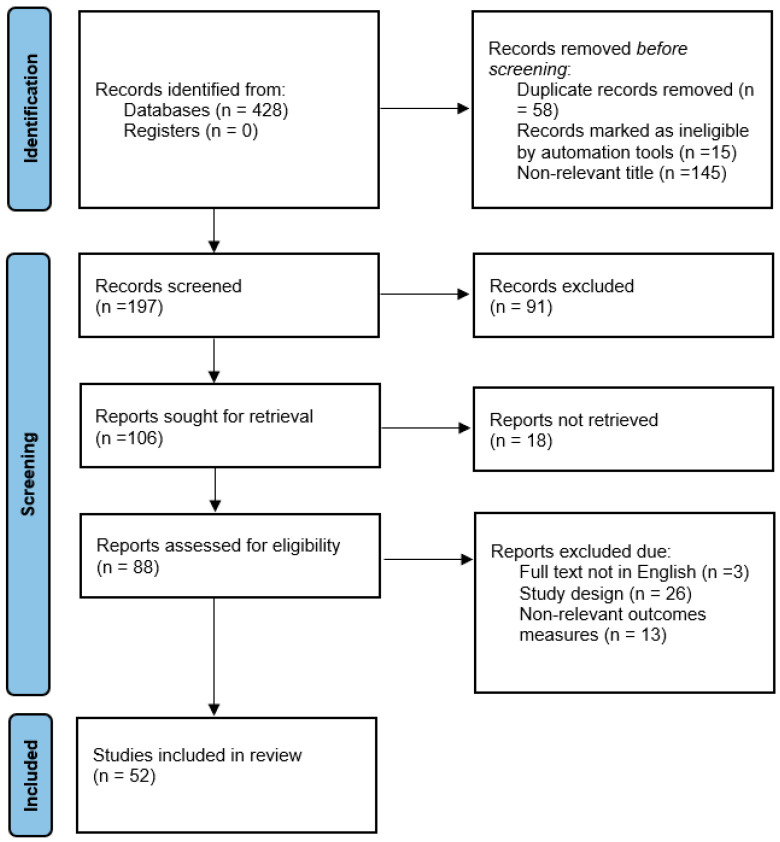
Flowchart summarizing the literature inclusion process.

To identify the available literature addressing our focused questions, a preliminary search was conducted on MEDLINE (PubMed) without any limitations in terms of study design, publication year, or language. The keywords used in the electronic search were as follows: “periodontitis”, “adverse pregnancy outcomes”, “periodontal diseases”, “preterm birth”, and “low birth weight”. All search results were reviewed by the two authors, and only relevant case–control, cohort, and randomized control studies were included. Articles were excluded if they were non-relevant, duplicates, and/or published in languages other than English. Overall, 52 articles discussing the relationship between periodontal diseases and adverse pregnancy outcomes (APOs), the role of specific pathogenic bacteria in the pathophysiology of APOs, and outcomes of periodontal therapy in reducing the risk of APOs were extracted as depicted in the flowchart in Figure 1.

## 3. Infection, Inflammation, and Adverse Pregnancy Outcomes

Infections are strongly associated with PLBW and, specifically, vaginal and urinary tract infections [15,17]. Whether through lower genital tract access or through the bloodstream, pathogens with their endotoxins enter the uterus, infect the amniotic fluid, and potentially cause chorioamnionitis, miscarriage, an early fetal membrane rupture, and preterm labor and birth [10]. Pathogenesis is the bacterial activation of fetal membranes followed by the production of inflammatory cytokines, chemokines, and growth factors as a result of maternal immune response stimulation. Consequently, elevated maternal and fetal cortisol levels, MMPs, and prostaglandins can not only induce contractions and preterm delivery but also restrict fetal growth and put the fetus in distress [18]. Distant organ infections can also affect the gestation period, including malaria, diarrhea, and respiratory tract infections of viral origin [15,17]. Furthermore, evidence suggests similar sequelae for maternal–fetal unit infections and subsequent consequences because of bacterial vaginosis occurring as a result of distant, low-grade oral infections [17]. Offenbacher et al. were the first to document a relationship between preterm birth and periodontal diseases in a case–control study of humans. They concluded that mothers who delivered preterm low-birth-weight infants had significantly worse periodontal disease status in comparison with mothers in a control group who completed a full-term gestational period [19]. This study was followed by a study in which the same authors found a significant increase in the prevalence of preterm births (<28 weeks) as the periodontal status of the mother continued to deteriorate [20].

## 4. Pro-Inflammatory Mediators and Adverse Pregnancy Outcomes

The physiologic induction of labor is associated with peak levels of several inflammatory mediators such as prostaglandin E2, IL-1β, TNF-⍺, and many others. As a result of periodontal pathogen influx and the dissemination of their endotoxins, an inflammatory response is triggered, activating inflammatory cell recruitment and the production of pro-inflammatory cytokines. It was hypothesized that this inflammatory cascade spreads systemically through the vasculature, potentially inducing labor prematurely. Several studies have tested this hypothesis and attempted to measure the concentration of pro-inflammatory mediators using gingival crevicular fluid (GCF) samples as a non-invasive screening tool in comparison with its levels in the blood plasma of pregnant women and amniotic fluid [21].

### 4.1. Interleukins

As a result of periodontal pathogen invasion, a series of inflammatory cytokines and bioactive molecules are released locally and systematically and are expressed in different body fluids such as GCF, the bloodstream, and amniotic fluid [22]. While these cytokines are expressed as a result of an inflammatory response, their levels are also expressed as a part of pregnancy. However, any disruption in the balance can result in prematurely induced labor. Upregulated levels of IL-6 as a result of periodontitis have been shown to be increased in the maternal–fetal complex, negatively impacting the fetal membranes and potentially causing preterm labor [22]. In fact, some studies suggest utilizing these cytokines as a diagnostic tool to evaluate the risk of delivering prematurely [23]. Circulating macrophages produce and release ILs, specifically IL-1β, which has been detected in amniotic fluid surrounding the fetus, and its concentration can triple by the end of the third trimester of pregnancy. Other studies using the GCF as a less invasive diagnostic marker reported that mothers who delivered preterm had significantly greater concentrations of IL-2, IL-6, and IL-10 in their GCF samples compared with samples obtained from mothers who completed full-term pregnancies [22,24].

### 4.2. Prostaglandins

During the normal course of pregnancy, the maternal body gradually and continuously releases prostaglandins, specifically PGE2 and PGF2. Toward the end of the third trimester, prostaglandins reach critical levels, thus physiologically initiating intra-uterine wall contractions and labor. It is well documented that any pathologic disruptions in prostaglandin levels prior to the end of the full term (similar to those occurring as a result of disseminated infections, as in chronic periodontitis) can induce labor prematurely or even result in abortion [23]. Moreover, prostaglandin inhibitors are used to delay and arrest early-onset labor symptoms. Upregulated PGE2 levels are associated with worse periodontal surrogate parameters, such as probing depth and clinical attachment levels [24]. Studies have consistently reported a dose-dependent positive relationship between PGE2 levels in collected GCF samples and the severity of periodontal destruction in mothers who also happened to be more likely to deliver preterm [23].

### 4.3. C-Reactive Protein

As a result of acute infection and in response to the influx of other pro-inflammatory mediators, including interleukin-1β, tumor necrosis factor-alpha (TNF-⍺), and prostaglandins, the liver produces C-reactive protein, which is a well-known inflammatory marker. Because of the chronic nature of the disease, elevated CRP levels are consistently observed in male and non-pregnant subjects diagnosed with periodontitis. Only a few studies have examined the association between CRP levels in pregnant women and periodontitis [25]. One study used blood samples to measure plasma CRP levels in 35 periodontally compromised subjects versus 66 periodontally healthy individuals. Excluding smoking and diabetes, along with other confounding factors, the results showed a 65% increase in plasma CRP levels in samples with periodontitis compared with healthy controls. While several limitations exist within the study, including a small sample size and a lack of negative controls, it is feasible to suggest that both C-reactive protein and periodontitis could be associated with adverse pregnancy outcomes. However, further investigation is needed [25]. A later study conducted in 2018 revealed the positive impact of nonsurgical periodontal therapy in reducing plasma CRP levels in pregnant women with periodontitis. However, the relationship between CRP and adverse pregnancy outcomes has yet to be explored [26].

## 5. The Relationship between Periodontal Disease and Adverse Pregnancy Outcomes

In the last three decades, numerous studies have investigated the possible relationship between periodontal disease and adverse pregnancy outcomes, as summarized in Table 1. The relationship may be explained by two mechanisms: the first is the direct translocation of periodontal pathogens to the fetoplacental unit, such as *Porphyromonas gingivalis*, *Fusobacterium nucleatum*, *Prevotella intermedia*, *Aggregatibacter actinomycetemcomitans* (A.A), and *Treponema denticola*, and the second being the indirect effect of inflammatory mediators, such as interleukin-1 (IL-1), IL-6, IL-8, tumor necrosis factor-alpha (TNF-α), and prostaglandin E2 (PGE2), all of which may trigger a systemic reaction. In response to this trigger, the liver upregulates and disseminates C-reactive protein and fibrinogen, both contributing to inflammation [27].

Early case–control studies failed to demonstrate a robust relationship between periodontal disease and PLBW [28,29]. On the contrary, a study conducted by Moreu et al. in Spain revealed a statistically significant positive association between increased maternal probing depth and PLBW. They considered maternal periodontal disease a risk factor for PLBW but failed to correlate it with preterm delivery [18].

**Table 1:** The relationship between periodontal disease and adverse pregnancy outcomes.

Author, Year	Study Design	Sample Size	Association
Cobos et al., 2022 [30]	Prospective cohort	102 subjects	No significant association between periodontal diseases and the incidence of preterm labor or low birth weight.
Giguère et al., 2016 [31]	Prospective cohort	273 subjects	A significant association between periodontal diseases was reported with preeclampsia only but not with spontaneous preterm birth.
Ardakani et al., 2013 [32]	Case–control	88 subjects	Mothers who delivered low-birth-weight infants had worse gingival health and deeper periodontal pockets.
Mannem et al., 2011 [33]	Case–control	104 subjects	Duration of pregnancy is affected by periodontal health status.
Khader et al., 2009 [34]	Case–control	148 subjects	The severity of periodontal diseases in mothers increased the odds of preterm low-weight births.
Mumghamba et al., 2007 [35]	Retrospective case–control	373 subjects	Periodontal diseases, among other factors, are not considered significant factors for preterm and low-weight births.
Skuldbøl et al., 2006 [15]	Case–control	21 women experienced preterm labor; 33 women experienced term labor	No association was found between periodontal disease and preterm labor.
Moreu et al., 2005 [18]	Observational	96 subjects	Periodontal disease is a significant risk factor for low birth weight but not for preterm delivery.
Moliterno et al., 2005 [36]	Case–control	151 subjects	Periodontal disease is a risk indicator for low birth weight.
Cruz et al., 2005 [37]	Case–control	302 subjects	A positive association between periodontal diseases and low birth weight.
Moore et al., 2004 [38]	Prospective	3738 subjects	No association was found between periodontal disease and preterm labor or low birth weight
Davenport et al., 2002 [29]	Case–control	236 cases 507 controls	No association was found between periodontal disease and preterm labor or low birth weight.
Offenbacher et al., 2001 [20]	Prospective	812 subjects	Prevalence of birth at <28 weeks was 1.1% in periodontally healthy mothers, 3.5% in mothers with mild periodontal disease, and 11.1% in mothers with moderate-to-severe periodontitis.
Offenbacher et al., 1996 [19]	Case–control	124 subjects	Periodontal disease is a significant risk factor for preterm labor (PTL), preterm rupture of membranes (PROM), and consequently, preterm low-birth-weight infants (PLBW).

## 6. Influence of Hormonal Changes during Pregnancy on Oral Microflora

Throughout pregnancy, a series of hormonal and metabolic fluctuations ultimately have a systemic influence on both cellular and immunologic levels to accommodate and adapt to the demands of the growing fetus. Governed by changes in sex hormones such as estrogen, progesterone, and gonadotropin, cellular changes are expressed in the form of cytoplasmic swelling in endothelial cells, an increased platelet count, enhanced intra-vascular neutrophils adhesion, enhanced vascular permeability, and increased risk of small thrombus formations [39]. On a periodontal tissue level, the impact of estrogen and progesterone manifests as an altered connective tissue cell turnover rate, an impaired vascular response, and increased permeability in the gingival vasculature, creating a pathway for periodontal pathogens to leak through and invade the circulation to establish infections in fetal–placental units [39]. Furthermore, modifications to the maternal hormonal profile during pregnancy trimesters can alter immune cell maturation and activation against infectious organisms [40]. Several studies have reported reduced maternal peripheral lymphocyte activation when subjected to antigens, and when compared with non-pregnant or male subjects, altered maternal estrogen and progesterone levels enhanced and suppressed prostaglandin E2 and interleukin-1β, respectively [41].

One proposed hypothesis discusses the link between elevated maternal hormonal levels and an increased number of periodontal pathogenic bacteria, which could be influenced by direct and indirect pathways, together facilitating the entrance and multiplication of pathogenic bacteria. This was demonstrated by Gibbons in the 1960s; pathogens, specifically *P. intermedia* and *P. gingivalis*, directly utilize these elevated hormones to produce vitamin K, a vital prerequisite for their bacterial growth [42], while greater hormone levels can indirectly shift gingival clinical parameters toward increased probing depth and a greater gingival crevicular fluid amount and rate, affecting the quality and quantity of marginal gingival keratinization and lowering the immune response [43]. A study conducted in 2010 attempted to detect periodontal pathogens in pregnant women, and a significant increase in bacterial counts, with peak levels during the second and third trimesters of pregnancy, was observed [34]. Furthermore, greater progesterone levels measured from salivary samples were significantly associated with *P. gingivalis* bacterial counts [44].

## 7. Gestational Diabetes Mellitus, Periodontal Diseases, and Adverse Pregnancy

Gestational diabetes mellitus (GDM), a well-known complication of pregnancy, can be defined as impaired or complete dysglycemia diagnosed during the gestational period. It has been reported that GDM may increase the incidence of adverse pregnancy outcomes, including gestational hypertension, miscarriage, and impaired fetal growth and development, as well as the progression of diabetes and obesity, even after childbirth. In a systematic review summarizing the findings of cross-sectional and case–control studies, mothers who presented with GDM were also found to have worse periodontal conditions, which can be observed when comparing clinical parameters such as the probing depths and clinical attachment levels of healthy and diseased individuals [45]. In fact, 65% of mothers diagnosed with GDM were also diagnosed with or had known cases of periodontitis. Furthermore, it was observed that the presence of GDM is associated with moderate-to-advanced periodontal diagnoses, manifesting in approximately 37.5% and 23% of mothers classified as stage II and stage III, respectively. With poor glycemic control, it has been proposed that a shift in oral microbiota takes place, changing from symbiosis into dysbiosis [46]. Based on an observational study comparing mothers suffering from both GDM as well as periodontitis with another group of healthy mothers with or without existing periodontal disease, those who suffered GDM were reported to have a significant prevalence of premature births; despite this, almost fifty percent of those infants were classified as large when compared with infants delivered by healthy mothers with similar gestational ages [46].

## 8. Periodontal Pathogens Associated with Adverse Pregnancy Outcomes

**Figure 2:**
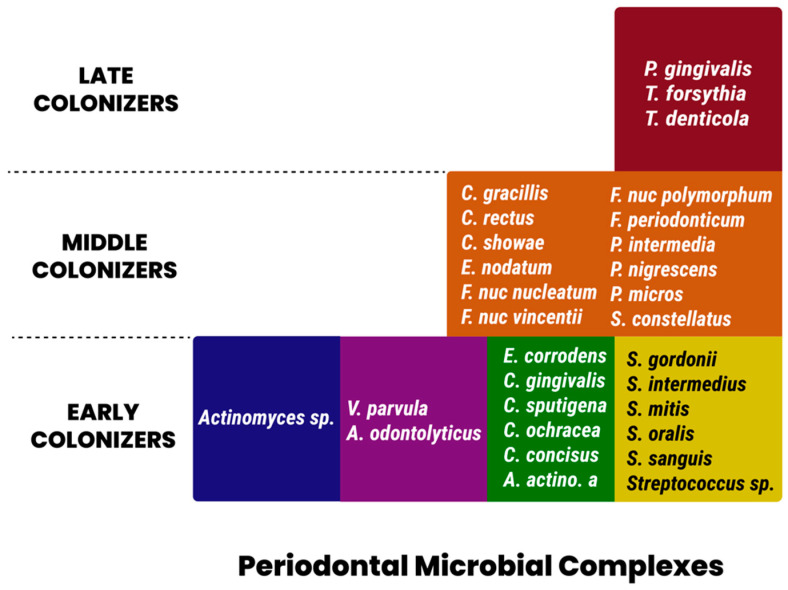
Periodontal pathogen complexes.

In 1998, Socransky et al. [47] described five major complexes that were consistently observed in plaque samples using whole-genomic DNA probes in addition to checkerboard DNA-DNA hybridization. These complexes were color-coded as red, orange, green, yellow, and purple. Members of the red complex consisted of *Bacteroides forsythus*, *Porphyromonas gingivalis*, and *Treponema denticola*. This complex is strongly associated with clinical measures of periodontal disease, specifically pocket depth and bleeding upon probing [38]. The species *P. intermedia*, *F. nucleatum*, *P. nigrescens*, *Campylobacter rectus*, and *Campylobacter showae* and *gracilis* comprised the orange cluster. Similar to the red complex, these species were found to be significantly associated with unfavorable deep periodontal pocket depths. Furthermore, members of the orange complex were found to be present in non-periodontitis-related infection sites [39]. A preponderance of *Streptococcal* species in the yellow complex, as well as, primarily, *Capnocytophaga* in the green complex, are associated with periodontal health and serve as early colonizers of dental plaque, potentially facilitating the adhesion of virulent, pathogenic red or orange complex communities. Despite its association with rapidly destructive incisor–molar-pattern periodontal diseases, *A. actinomycetemcomitans* did not cluster with the pathogenic red or orange complex organisms. Socransky also evaluated the relationship between these complexes within periodontal pockets and reported the frequent co-presence of both red and orange complex members in the same periodontal pocket. He further concluded a strong positive association between the presence of both red and orange complexes and deeper periodontal pockets. Figure 2 summarizes the periodontal pathogen complexes [47].

Recently, a trend arose in examining the influence of members of the orange and red complexes (those strongly associated with severe periodontal diseases) on maternal infections and subsequent related complications, as summarized in Table 2. Mothers with low levels of protective IgG against orange and red complex organisms had high fetal IgM levels, indicating a systemic distribution of maternal oral flora, which, in turn, elicited an antibody response in the fetus and is associated with a high pre-maturity rate of 66.7% [48]. Lin et al. measured the microbial levels of red complex species along with *A.A* bacteria in pregnant women. The findings included a tendency toward increased bacterial loads of those species as pregnancy progresses from the twenty-second week in mothers with premature fetuses versus those who delivered full-term. Furthermore, a significant 2.4-fold increase in the prevalence of *A.A* bacteria occurred between early and late pregnancy trimesters in preterm versus full-term mothers [49].

### 8.1. Porphyromonas Gingivalis

*P. gingivalis* is a coccal and/or rod-shaped, Gram-negative, anaerobic, non-motile bacteria that is a keystone pathogen of periodontal disease initiation [56]. *P. gingivalis* is known for its ability to produce degrading gingipains and collagenolytic and proteolytic toxins, and it is unique in possessing many intricate mechanisms to navigate and colonize the fetoplacental unit, contributing to the onset of many adverse pregnancy outcomes [53]. Following the invasion of maternal tissues by *P. gingivalis*, cellular changes such as increases in neutrophil influx, the inhibition of natural killer and T-cells, and the proliferation and alteration of extra-villous trophoblasts (vital to the growth and development of placenta) take place in the placenta [54]. As a result, a higher risk of tissue injury, improper or altered placenta formation, and reduced placental size might occur [50]. Furthermore, *P. gingivalis* can upregulate up to twofold the production of pro-inflammatory cytokines such as IL-6, IL-17, IL-1β, and TNF-⍺ via interactions with maternal cellular signaling molecules and receptors [55]. Alterations in the production of acute-phase reactive proteins such as C-reactive protein (CRP) and Pentraxin-3 (PTX3) are another virulent characteristic of *P. gingivalis* that might put the mother at higher risk of developing preeclampsia, gestational diabetes, spontaneous miscarriage, or preterm labor [57,58].

### 8.2. Fusobacterium Nucleatum

*Fusobacterium nucleatum (F. nucleatum*) is a Gram-negative rod/spindle-shaped anaerobic species found most frequently in subgingival microbial communities. Recently, it was also detected in amniotic fluid and found to be the organism exclusively responsible for preterm deliveries with intact fetal membranes [59]. With its unique virulence mechanisms, such as fibroblast activation protein 2 and fusobacterium adhesion A, this bacterium is capable of invading placental tissues, making it a mutual pathogen found in both preterm births and periodontitis cases. Moreover, in a study conducted on mice, *F. nucleatum* was capable of activating Toll-like receptors, resulting in inflammatory responses in placentas [60]. Several studies have been conducted to investigate the relationship between the two diseases. In pregnant mice injected with *F. nucleatum*, it demonstrated hematological invasion capabilities in endothelial cells and blood vessels, amniotic fluids, and placental tissues and was associated with adverse pregnancy outcomes [50,52]. Similar results have also been found in humans [51].

### 8.3. Campylobacter Rectus

As a result of metastatic pathogen infections from the periodontium in the placenta, elevated levels of *Campylobacter rectus*-associated IgM frequently found in preterm infants not only could implicate a causal association between this pathogen and preterm delivery, as well as PLBW, but also was associated with potentially life-threatening fetal manifestations. Recent studies found an association between this anaerobic, Gram-negative motile species and placental inflammation, decidual hyperplasia, and elevated levels of interferon-gamma that could result in fetal brain damage [12]. It is important to note that these findings were confirmed in mice models, and further investigations should be carried out to compare adverse outcomes associated with this pathogen in humans.

## 9. Outcomes of Periodontal Therapy Interventions in Reducing Adverse Pregnancy Outcomes

To investigate causality, multiple studies have attempted therapeutic interventions at different trimesters aiming to eliminate pathogens and determine if the risk of adverse pregnancy outcomes would be reduced. Preliminary results from prospective and case–control studies suggested the positive influence of treating periodontal diseases in lowering the incidence of PLBW [61]. In 2005, a study conducted by Lopez et al. addressed the outcomes of periodontal therapy in pregnant women suffering from gestational-associated gingivitis in reducing PLBW rates, and they found significantly reduced incidence rates of 2.14% versus 6.71% in the test and control groups, respectively [61]. Several studies adopted similar methodologies to evaluate the outcomes of periodontal therapy carried out either during the gestational period or treatments performed following delivery in mothers suffering periodontitis [62,63]. These studies reported that nonsurgical periodontal therapy during pregnancy was effective in reducing the incidence of PLBW in mothers. It is noteworthy to mention that these studies were single-centered in nature and, therefore, of a smaller sample size. Offenbacher et al. [64] conducted a multi-center, randomized control trial that included 1760 pregnant women randomized to receive periodontal therapy either during pregnancy or following delivery, and they found no significant difference in the incidence of PLBW between the two groups. Furthermore, neonatal morbidity and mortality factors such as sepsis, intra-ventricular hemorrhages, respiratory distress syndrome, and other congenital abnormalities, as well as other adverse pregnancy outcomes, including stillbirths and spontaneous abortion, were investigated as secondary outcomes, with no reported statistically significant difference. There was, however, a significant deterioration in the periodontal status of patients who received the treatment after delivery [64].

Because of study design heterogenicity, potential improper randomization, a lack of control groups, and low-quality evidence, this review was not able to clearly provide a statement regarding the impact of mechanical periodontal therapy in reducing the incidence of adverse pregnancy outcomes. While further studies are needed to support this hypothesis, a positive correlation should be sufficient to initiate and implement a maintenance program for pregnant women [65].

## 10. Discussion

There is conflicting evidence from randomized-control-trial-based systematic reviews discussing the relationship between the outcomes of periodontal therapy and adverse pregnancy outcomes. While some studies found a favorable and significant difference between periodontally treated groups in comparison with control groups [50], others have reported otherwise [64]. It is noteworthy to mention the following potential limitations when addressing this relationship:

First, periodontitis cannot be treated through single or even multiple intervention sessions. To bring the patient back to “periodontal health” or a “reduced stable periodontium”, surgical interventions may be needed to eliminate subgingival pathogens and reduce periodontal pockets, thus improving clinical attachment levels [57]. Since surgical procedures of a non-emergency nature are considered elective procedures and are usually limited to the second trimester, they are often postponed until after delivery.

Second, the outcomes of periodontal treatments can be influenced by confounding factors such as hormonal changes during pregnancy, naturally elevated pro-inflammatory markers and cytokines, and other factors such as age, smoking, obesity, gestational diabetes, and socioeconomic status, which were not always controlled for in the reported literature [64].

Finally, despite some studies finding an influential relationship between periodontitis and adverse pregnancy outcomes, its treatment may not necessarily reduce the risk of adverse pregnancy outcomes [66]. Bacterial vaginosis, for instance, was considered a contributing factor to preterm delivery. However, McDonald et al. reported very little benefit from systemically administering antibiotics to eradicate bacterial infections in reducing the likelihood of PBLW [67]. Therefore, it is reasonable to assume that intra-conception interventions are potentially too late to have a significant impact [68], and it may be necessary to establish and promote periodontal health in the preconception period.

Exploring exposures such as peri-implantitis and their treatment with or without adjunctive use of diode lasers would be an interesting addition to future studies looking at this relationship [69].

## 11. Conclusions

Taking into consideration the pathogenic mechanisms of periodontitis, including the infectious nature of the disease, the upregulation of pro-inflammatory cytokines, host responses to bacterial infections, and physiologic changes occurring during pregnancy, it is reasonable to consider periodontal disease an important yet modifiable risk indicator for adverse pregnancy outcomes.

While several studies have found a positive correlation between periodontal diseases and adverse pregnancy outcomes, this correlation remains weak because of inconsistent methodologies in the reported studies. Most reported studies assessing periodontal disease and adverse pregnancy outcomes were case–control study designs and/or used animal models. Conversely, prospective cohort studies with adequate sample sizes found no such association. Periodontal treatment interventions, both surgical and nonsurgical, are necessary for eliminating periodontal pathogens and may improve pregnancy outcomes. However, the timing of treatment is also important, as evidence from randomized trials suggests that nonsurgical periodontal interventions at later stages of pregnancy yield no significant impact in reducing the incidence of adverse pregnancy outcomes. A possible reason is that periodontal pathogens may have already colonized and infected the amniotic fluid. Moreover, we recommend promoting oral hygiene and considering periodontal evaluation as a part of preconception examination routines. We may summarize our conclusion in the following points:

- Periodontal disease may be a modifiable risk indicator.
- More robust study designs are needed to further investigate the relationship.
- Nonsurgical interventions may be more beneficial in earlier stages of pregnancy.

## Figures and Tables

**Table 2:** Periodontal pathogens associated with adverse pregnancy outcomes.

Author, Year	Study Design	Sample	Periodontal Pathogens	Conclusions
Buduneli et al., 2005 [12]	Case–control	Humans	*P. gingivalis*, *P. intermedia*, *P. nigrescens*, *A.A*, *Streptococcus intermedius*, *F. nucleatum*, *Peptostreptococcus micros*, *C. rectus*, *Eikenella corrodens*, *Selenomonas noxia*, and *S. intermedius*	*P. micros* and *C. rectus* were associated with PLBW.
Garcia et al., 2019 [50]	Case–control	Animals	*F. nucleatum*	Omega-3 fatty acids suppress inflammatory responses elicited by bacteria, making them potentially effective in reducing adverse pregnancy outcomes.
Han et al., 2010 [51]	Case report	Animals	*F. nucleatum*	*F. nucleatum* can translocate from the oral to the uterine environment.
Han et al., 2004 [52]	Case–control	Animals	*F. nucleatum*	The hematologic transmission capabilities of *F. nucleatum* could induce adverse pregnancy outcomes.
Chopra et al., 2020 [53] Reyes et al., 2017 [54]	Review	N/A	*P. gingivalis*	The pathogenesis of *P. gingivalis* is associated with adverse pregnancy outcomes: - Maternal/fetal tissue alterations via the action of virulence factors, surface adhesion molecules, and enzymes; - Enhanced production of cytokines, oxidative stresses, and acute-phase proteins; - Elevated fetal adrenal cortisol levels.
Ao M et al., 2017 [55]		Animals	*P. gingivalis*	- The LPS of *P. gingivalis* was capable of pro-inflammatory cytokine upregulation; - Translocation capabilities of *P. gingivalis* in utero.
Lin et al., 2007 [49]	Case–control	Humans	*P. gingivalis*, *Tannerella forsythia*, *Prevotella intermedia*, and *Prevotella nigrescens*, *A.A*	- Statistically significant higher levels of all bacteria associated with preterm births in comparison with full-term births; - A 2.4-fold increase in levels of *A.A* bacteria in preterm deliveries as the pregnancy progresses until after birth when compared with full-term deliveries.

## Data Availability

Not Applicable.
